# Ligand Binding and Circular Permutation Modify Residue Interaction Network in DHFR

**DOI:** 10.1371/journal.pcbi.0030117

**Published:** 2007-06-15

**Authors:** Zengjian Hu, Donnell Bowen, William M Southerland, Antonio del Sol, Yongping Pan, Ruth Nussinov, Buyong Ma

**Affiliations:** 1 Department of Biochemistry and Molecular Biology, Howard University College of Medicine, Washington, District of Columbia, United States of America; 2 Bioinformatics Research Project, Research and Development Division, Fujirebio, Tokyo, Japan; 3 Basic Research Program, Science Applications International Corporation—Frederick, Inc., Center for Cancer Research Nanobiology Program, National Cancer Institute—Frederick, Frederick, Maryland, United States of America; 4 Sackler Institute of Molecular Medicine, Department of Human Genetics and Molecular Medicine, Sackler School of Medicine, Tel Aviv University, Tel Aviv, Israel; Indian Institute of Science, India

## Abstract

Residue interaction networks and loop motions are important for catalysis in dihydrofolate reductase (DHFR). Here, we investigate the effects of ligand binding and chain connectivity on network communication in DHFR. We carry out systematic network analysis and molecular dynamics simulations of the native DHFR and 19 of its circularly permuted variants by breaking the chain connections in ten folding element regions and in nine nonfolding element regions as observed by experiment. Our studies suggest that chain cleavage in folding element areas may deactivate DHFR due to large perturbations in the network properties near the active site. The protein active site is near or coincides with residues through which the shortest paths in the residue interaction network tend to go. Further, our network analysis reveals that ligand binding has “network-bridging effects” on the DHFR structure. Our results suggest that ligand binding leads to a modification, with most of the interaction networks now passing through the cofactor, shortening the average shortest path. Ligand binding at the active site has profound effects on the network centrality, especially the closeness.

## Author Summary

The cooperative movements within a protein concerning the binding and dissociation of the reactants and products could be important for protein function. Communication among the various parts of an enzyme can be achieved by the networks connecting amino acids through peptide backbone connections and nonbonded amino acid contact. We used dihydrofolate reductase (DHFR), a clinically important enzyme, as an example to explore the effects of amino acid communication on protein functions. We found that the peptide chain itself is an efficient “telephone wire” to transfer the communications. Breaking the telephone wire (peptide chain) at different points leads to differentiated behavior near the enzyme active site. The important points to keep the peptide chain communication are coupled with the place where protein folding occurs. On the other hand, ligand binding to the enzyme active site provides a “short cut” to the communication networks, with most of the interaction networks now passing through the added ligand and shortening the average communication path. We considered the short cuts to be “network-bridging effects” in the protein structure. The enzyme active site is the place where the short cut has the most dramatic effect in modifying protein communication networks.

## Introduction

Extensive experimental studies of dihydrofolate reductase (DHFR) have provided rich data toward the structure–function relationship in proteins. Escherichia coli DHFR is a 159–amino acid, monomeric, two-domain protein that is well characterized in terms of structure and function. DHFR catalyzes the reduction of 7,8-dihydrofolate (DHF) to 5,6,7,8-tetrahydro-folate (THF) using the reducing cofactor nicotinamide adenine dinucleotide phosphate (NADPH). DHFR is a clinically important enzyme and is the target of a number of antifolate drugs.

Experimental kinetic analysis of various DHFR permutants has identified several loop regions important for catalysis [1,2]. Figure 1 illustrates the loop locations. The Met-20 loop (residues 10 to 23) directly controls the ligand binding to DHFR. The FG loop (residues 116 to 121) is behind the Met-20 loop. There is a network of hydrogen bonds connecting the Met-20 loop and the FG loop. The third GH loop (residues 142 to 149) is in contact with both the Met-20 and the FG loops. Based largely on the conformation of the Met-20 loop [3], the three states of the enzymatic reaction process (binding and release of cofactor, substrate, and product) can be defined using available crystal structures (Figure 1). In the open state (Figure 1, green ribbon) the Met-20 loop is flipped away from the binding site. In the closed state (Figure 1, blue), the Met-20 loop packs against the cofactor and seals the active site. In the occluded state, the Met-20 loop blocks the binding of the cofactor in the pocket. Simulations of the closed state also indicate changes in the other side of the binding pocket, in the helix region (residues 44 to 50), which binds the cofactor. Loop region 64–71, which contacts the helix, also presents large fluctuations.

**Figure 1:**
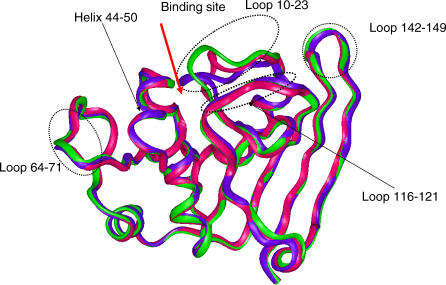
Illustration and Superposition of the Three States of DHFR Red indicates occluded; green, open; and blue, closed. Five regions (loop10–23, helix 40–45, loop 64–71, loop116–121, and loop 142–149) near the active sites are highlighted.

The cooperative movements of these loops couple with the overall dynamics of the protein concerning the binding and dissociation of the cofactor, substrate, and product. The communications among the various parts of the DHFR can be achieved by residue interaction networks and through peptide backbone chain connections and nonbonded residue interactions. Agarwal et al. carried out genomic analysis of sequence conservation, kinetic measurements of multiple mutations, and theoretical calculations, observing that nonbonded residue interactions in DHFR form a network of coupled motions that are important for enzyme catalysis [4].

The effect of the peptide backbone chain connection on the protein dynamics is more complex, since chain connection is coupled with protein folding. Circular permutations of DHFR provide insight into chain connectivity, stability of the fold, and function. Circular permutation of a protein consists of connecting the native N- and C-termini covalently with a peptide linker and cleaving the peptide backbone at another specific site. Iwakura et al. have performed systematic circular permutation of the entire DHFR protein to investigate essential folding elements [5–8]. Other groups [9–11] have also circularly permuted the protein, selectively focusing on several permutations or cutting the backbone connection [11] to test fragment complementation. It was found that the peptide bonds in the protein could be grouped into two categories based on the effects of breaking the backbone connectivity. While cleavage of some peptide bonds results in less active variants or affects the enzyme function only slightly, suggesting that these make only minor contributions to the ability of the protein to fold, cleavage at certain other positions leads to a complete loss of the ability of the protein to fold. When such cleavage sites occurred sequentially in the primary sequence and formed a contiguous peptide segment, the region was named a “folding element,” which is crucial for a protein to be foldable [5–8]. Folding elements distribute throughout the sequence. It was proposed that a complete set of folding elements is necessary for a protein to fold [5]. By conducting a systematic circular permutation analysis in which the original N- and C-termini of a protein are connected by an appropriate linker and new termini are created sequentially, ten folding elements have been identified in E. coli DHFR, each of which contains two to 14 residues [5–8] (Figure 2). It was also found that although the positions of the folding elements do not appear to correspond to the secondary structure motifs or to binding sites, almost all of the amino acid residues known to be involved in early folding events of DHFR are located within the folding elements, suggesting a close relationship between the folding elements of a protein and early folding events.

**Figure 2:**
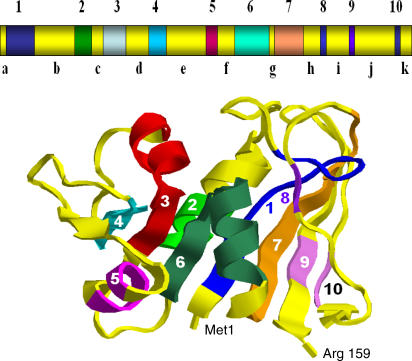
A Drawing of the Tertiary Structure of E. coli DHFR Taken from the Protein Data Bank and the Locations of the Proposed Folding Elements [5] Four α-helices and eight β-strands are shown as ribbons. Proposed folding elements are light blue (1: Ser3~Ile14), green (2: Trp30~Leu36), red (3: Val40~Ser49), cyan (4: Arg57~Ser63), magenta (5: Glu80~Ala83), greenish-blue (6: Ile91~Leu104), orange (7: Ala107~Glu118), purple (8: His124~Phe125), and light purple (9: Val136~Ser138; and 10: Glu154~Ile155)*,* respectively. The regions outside the folding elements form amino acid segments a–k (in yellow). The positions of the N- and C-termini are also indicated. Also see Table 1.

**Table 1:**
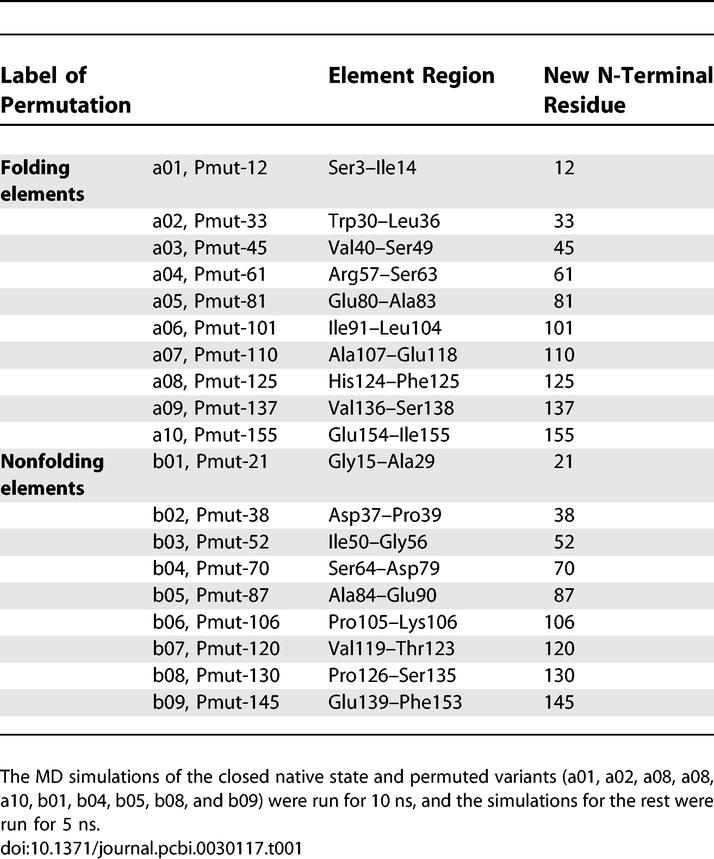
An Overview of the Performed Simulations

In order to delineate the complex relationship among chain connectivity, protein folding, coupled networks, and catalysis by DHFR, we have carried out a systematic network analysis and molecular dynamics (MD) simulations of the native (closed state) DHFR and 19 of its circularly permuted variants. We first obtained average protein structures from MD simulations of the native DHFR and of the circularly permuted mutants. We investigated the relationship between small-world network behavior and chain connectivity using the crystal and MD average protein structures. Small-world network analysis of the protein structure uses graph theory to explore the bonded and nonbonded amino acid residue network. It was first used to identify key residues in protein folding, as these residues have high connectivity (betweenness) with respect to all possible network connections in the transition states of the protein structures [12]. The concept has been extended to the protein-folding process [13,14], the protein–protein interface [15], protein structure [16] and stability [17], protein dynamics [18], and key functional residues in enzymes [19]. By comparing the centrality of the residue interaction network, we found that ligand binding at the functional site has profound effects on the global network connections. Using the network connectivity index to distinguish between bonded and nonbonded connections, we found that breaking the chain connection in folding elements has a greater effect on active site loops than does breaking the chain in nonfolding regions. This leads us to conclude that the native sequence was selected to maximize the coupling between the protein fold and its functional dynamics. A folding–function interrelationship might particularly be the case for a fold like the DHFR, which currently has only been observed in the DHFR family, fulfilling a single function.

## Results/Discussion

### MD Simulations of Native DHFR and Circular Permutation Variants

#### Conformational fluctuations of native DHFR and circular permutation variants.

We first investigated the overall structural change of the native (closed state) protein as observed in the MD simulations by computing the root mean square deviations (RMSDs) between the initial structure and snapshots along the trajectory. The profile of the C_α_ RMSD trajectory of the native DHFR (Figure 3A; blue) resembles profiles obtained for stable protein structures. Initially, the C_α_ RMSD of the native DHFR increases rapidly to an average value of 1.7 Å, followed by a gradual rise to about 2 Å at 5 ns and remaining nearly constant in the next 5 ns (Figure 3B). In order to test the effect of prolonged MD simulations on the structural network analysis, we extended the simulations for ten selected circularly permuted variants (five from the folding group and five from the nonfolding group) for additional 5 ns (Figure 3B). There is no significant structural change for additional 5-ns simulation times for the permuted variants. The overall simulation time is 145 ns for the 19 tested circular permutants (Table 1); among these, in ten variants the peptide bond is broken in folding element regions, and in nine the peptide bond is broken outside the folding elements.

**Figure 3:**
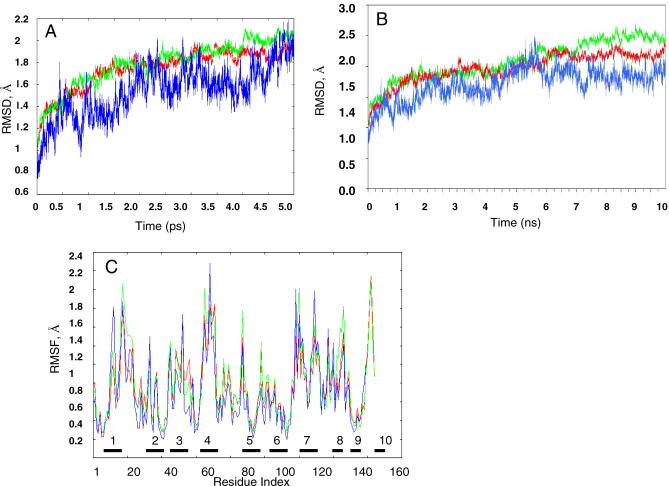
MD Trajectories (A) Comparison of the all-C_α_ atom RMSDs calculated from the MD trajectories for the folding element group, nonfolding element group, and the native DHFR at 300 °K during the 5-ns simulation (red: folding elements, green: nonfolding elements, blue: native DHFR). (B) Comparison of the all-C_α_ atom RMSDs calculated from the MD trajectories for five folding element variants (a01, a02, a08, a08, and a10), five nonfolding element variants (b01, b04, b05, b08, and b09), and native DHFR at 300 °K during the 10-ns simulations (red: folding elements, green: nonfolding elements, blue: native DHFR). (C) The comparison of RMSF for the folding element group, nonfolding element group, and the native DHFR averaged over 2.5- to 5.0-ns simulations (red: folding elements group, green: nonfolding elements group, blue: native DHFR). The blocks and numbers along the *x*-axis indicate the folding element region; see Figure 2 and Table 1 for reference.

The all-C_α_ RMSDs averages (with respect to the starting geometries) of the folding element and nonfolding element groups calculated from the MD trajectories are given in Figure 3A. Comparison of these suggests limited differences between the ten circularly permuted variants of the folding element group and the nine variants of the nonfolding element group during the simulations. Additional MD simulations up to 10 ns show that the permuted variants of the nonfolding element group have slightly higher fluctuation than the folding element group (Figure 3B). It appears that the break of the peptide bond outside the folding element regions leads to relatively high flexibility, whereas breaks occurring in the folding elements can be stabilized by the surrounding residues.

The comparison of the root mean squared fluctuation (RMSF) for each residue in the folding element group versus the nonfolding element group is shown in Figure 3C. The locations of the folding regions (labeled by numbers) and nonfolding regions (labeled by letters) on the structure are given in Figure 2 and are enumerated in Table 1. These regions are taken from the experimental study [5]. In the simulations, we observe that relatively smaller fluctuations occur in folding regions 1–7. Only regions 8–10 have relatively larger fluctuations during the simulation. The large fluctuations occur mostly outside the folding element regions, in regions b, d, e, f, h, and i. This is also observed in the comparison of the RMSF for the folding and nonfolding element groups (Fig 3C). While similar to the native DHFR, most of the large fluctuations occurred outside the folding element regions; in most cases, the RMSF values of the folding element group were smaller than the nonfolding element group. Again, the tightly packed environment of the folding elements stabilized the local region.

We did not observe unfolding in permutants with cuts in folded regions. Assuming that the experimental observations of enzymatic deactivations are caused by unfolding, there are two possibilities for our inability to capture the unfolding events. (1) None of the variants with cuts in folding elements is indeed able to fold into a native-like state. In such a case, our short 5-ns simulations of permutation variants in the folding elements are most likely trapped since we started the simulation with the native-like folded state. The additional 5-ns simulations are unable to change the scenario. (2) Some variants with cuts in the folding elements are still able to fold into native-like folds if helped by a chaperone, but without native enzymatic function due to effects on the active sites. It has been found that there is a close relationship between folding elements and the early folding site [5]. The systematic permutation experiments coupled folding and function, since a folding element was assigned to a continuous region when the cleavage abolished DHFR enzymatic function [5]. Therefore, it is possible that some permutation mutants, even with a folded native-like structure, may nevertheless lose function due to other reasons.

We stress that our simulations of the circularly permuted folding element group do not assess the folding ability of the systems with respect to either of the above two scenarios; rather, under the assumption that the permutants are able to fold, our simulations address effects of chain connection on the overall dynamics of protein. As seen in Figure 3C, comparison of the overall structural fluctuations from the MD simulations illustrates that we are unable to distinguish between variants with cuts in folding or in nonfolding elements. In the next section, we compare the structural changes, focusing on loop movements.

#### Comparison of three states of native DHFR and average structure of circular permutation variants.

Experimental kinetic analysis of various DHFR permutants has identified several loop regions important for catalysis [1,2], such as the Met-20 loop, the FG loop (residues 116 to 132), and the third GH loop (residues 142 to 149).

We systematically compared the structural variations for the three native states (i.e., the open, the occluded, and the closed), the average structure from the native (closed) state simulation, and the two average structures from the simulations of the circular permutation variants of the folding and nonfolding elements (Table 2). As shown in Figure 1, the difference between the three native states is in loop 10–23, with an average displacement of 1.7 Å between the closed and open states, 2.6 Å between the occluded and closed states, and 2.1 Å between the occluded and open states. The average structure from the simulation has a large deviation from the three crystal structures (the closed, open, and occluded states), and has an additional large change in loop 64–71 (average structure of wild-type DHFR [wild-ave]; Table 2). Using the closed state as a reference, the group of permutation variants with cuts in folding elements has a slightly smaller structural variation than that of the nonfolding element cutting group. The average structures of Pmut-45 (permutant with a cut at residue 45; a folding element group cut) and Pmut-87 (a nonfolding cut) have very small variations from the closed state crystal structure. When we use the open state crystal structure as a reference, we observe that Pmut-137 from the folding group and Pmut-106 from the nonfolding group have the least structural variation. When we compare with the occluded structure, we see that all average structures for permutant variants obtained from the MD simulations have large deviations in the range of 3–4 Å in the loop 10–23 region. In summary, the loop conformations in the MD average structures are more similar to the open or closed states than to the occluded structure. Examination of the geometric features of the average structures does not show any noticeable difference among the average structures for the native state, the folding region, and the nonfolding region cutting groups.

**Table 2:**
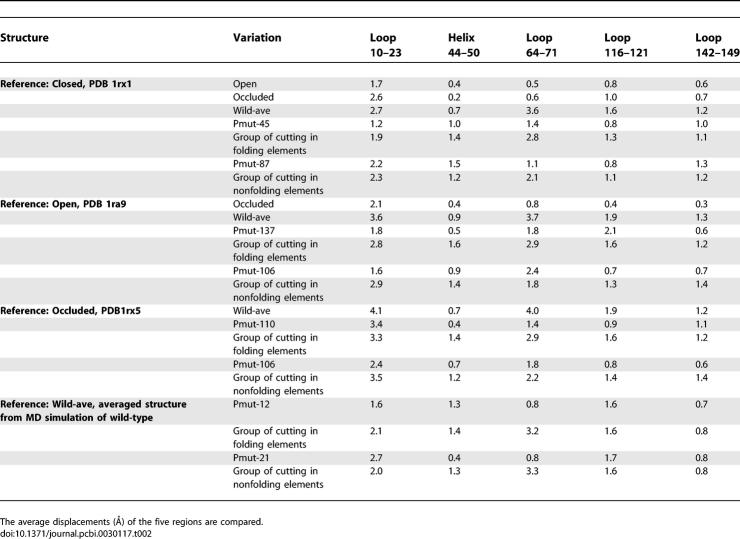
Structural Variation of the MD Average Structures for the Native DHFR and Its Circular Permutation Variants

### Changes in the Residue Interaction Network of Native DHFR by Ligand Binding and Circular Permutations

#### Cofactor binding and its network-bridging effects.

Network analysis of protein structures has shown that residues with high closeness values are likely to be functionally important, interacting directly with or close to the binding site [19]. Closeness centrality correlates more accurately with critical residues than betweenness [20]. The above studies imply that the protein active site is near or coincides with residues through which the shortest paths in the residue interaction network tend to go. Therefore, binding of a ligand to the active site could modify the residue interaction network. As a first step in our quest for a relationship between chain connectivity and functional site interaction network, we investigate the closeness changes upon a ligand binding to the DHFR active site.

The nodes of the residue interaction network of DHFR are the C_α_ of all residues. In the DHFR–cofactor complex, the cofactor skeleton is represented by the nitrogen and oxygen atoms because the C_α_ representation does not apply to organic molecules. We investigated the closeness change for the protein structure with and without the ligand. In Figure 4A, the blue line is the plot of the closeness of the apo form of DHFR (closed state), and the pink line represents the closeness for the DHFR–cofactor complex (closed state). As shown in Figure 4A, the cofactor binding has profound effects on the network centrality, especially the closeness of DHFR. We can see that all closeness peaks from residues 1–110 systematically increase after the cofactor binding, suggesting that most of the network interactions now pass through the cofactor, and that the average shortest path is shorter following cofactor binding. We call the effect of ligand binding on the protein residue interaction network the “network-bridging effect.”

**Figure 4:**
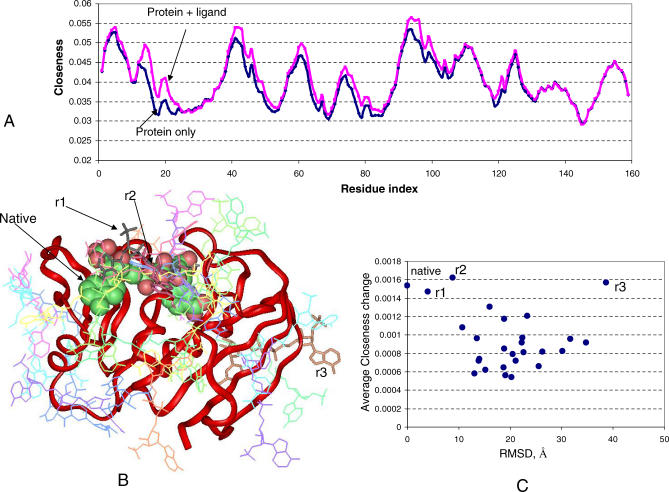
Network-Bridging Effect of Ligand Binding (A) Comparison of the closeness of native DHFR with and without cofactor binding. The blue line is the plot of the closeness of the apo form of DHFR, and the pink line is the closeness for the DHFR–cofactor complex. (B) Clusters of docking solutions from patch-docking. The native cofactor is represented as colored spheres, and r1 and r2 are also located in the native binding site, but with different orientation. r3 is away from the binding site. Other docking solutions scattered around the DHFR surface in different regions. (C) Average closeness change compared with DHFR after ligand binding into various surface patches; see Figure 3A.

In order to see if the network-bridging effect is unique to the protein–ligand interaction around the binding site, we examined the residue closeness change when the ligand is docked to different DHFR protein surface patches. In the docking study, we keep the ligand structure rigid. A total of 27 clusters were generated from the patch-docking process. In Figure 4B, the native ligand is shown as spheres, and the docked solutions are shown as lines and sticks (sticks for r1, r2, r3, see below; lines for all others). As can be seen in Figure 4B, the docked solutions distribute around the native binding site, as well as at other surface patches around the DHFR. The best docking result (ranked first in the solutions) locates in the native binding site, with 4 Å RMSD from the native ligand (r1, Figure 4B). Another cluster (ranking fifth among the docking solutions) has an 8.9 Å RMSD from the native ligand (r2; Figure 4B).

Depending on the locations of the docked ligand-binding sites, the network-bridging effect changes; and, among all docked solutions, the native bound conformation is within those with the largest closeness changes. Figure 4C plots the average closeness change upon ligand binding for the native and 27 docking clusters. The *x*-axis is the RMSD from the native ligand when superpositioning the DHFRs. Only three docking clusters caused closeness changes comparable with the native; two of them are located at the native binding site (r1 and r2; Figure 4B and 4C). The third, r3, is located near the strands formed by residues 1–15, which constitute one of the centers of the residue network of DHFR. Most other docking solutions away from the native binding site do not change the overall closeness pattern. Consistent with previous analysis of protein active sites that have high closeness values [19], here we show that ligand binding at the active site changes the overall closeness values significantly. This indicates that the native functional site of DHFR is sensitive to changes in the residue interaction network and to ligand binding at the active site, leading to the largest network-bridging effect.

The results point to future studies to systematically investigate ligand-binding effects in protein structure networks and the relationship to binding affinity. Our preliminary studies of 230 protein–ligand complexes confirm the general network-bridging effect, and suggest possible correlations of the change of closeness and ligand-binding affinities.

#### Network analysis of DHFR circular permutation variants: Chain connection and binding site closeness changes.

We systematically investigated the network centrality for the native DHFR and its circular permutation variants. First, we examined the crystal structure to obtain the betweenness of locations of important network residues and the locations of the cuts in the permutations. Agarwal et al. identified several key residues in DHFR enzymatic catalysis [4]. The locations of these residues are within the folding elements (for example, Phe31 and Met42) and the nonfolding elements (like G15 and G121, and Asp122). In general, these key residues and the cutting points within the folding elements have higher betweenness than the cutting points in the nonfolding group (Table 3). Analysis of the MD average structure also shows that the z-scores of centrality are higher for the folding element group. The results confirm the importance of the folding element regions in network interaction and protein folding. However, as to individual cutting, we do not see complete agreement, and not all residues are important for folding. Several residues have very low betweenness, and no shortest path goes through residue 102.

**Table 3:**
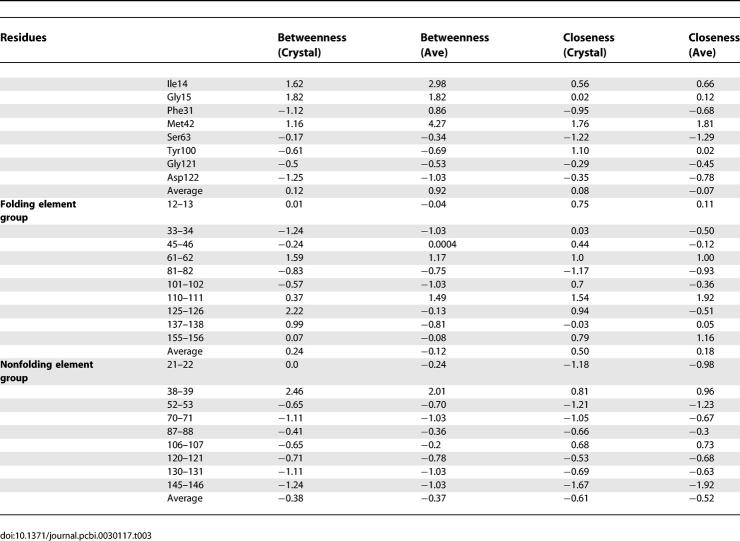
The z-Scores of the Centrality for Key Residues

Comparison of the betweenness of the crystal structure (closed state) with the MD average structure shows limited changes in certain areas (Figure 5A). The average structure has higher betweenness for residues 1–10, an area experimentally shown to be important in DHFR folding [5]. This area was also proposed to be important as an intramolecular chaperone to guide folding of other regions [21]. In the region around residue 125, the betweenness and closeness of the MD average structure drop sharply. This region is among the folding fragments (of residues 124–125), which also raises the question of the potential folding significance of the fragment. Early work using network analysis to identify the folding nucleus was based on a computed transition structure in protein folding [12], rather than on the crystal structure. Later, Paszkiewicz et al. published their study predicting viable circular permutants using network analysis based only on the crystal structure [22]. They found that in order to increase the match between predictions and experimentally determined folding regions, they needed to break the DHFR into two domains. In our study, we do not focus on the prediction of possible cutting points. Rather, we are interested in the effects of breaking chain connections on network properties near the active site.

**Figure 5:**
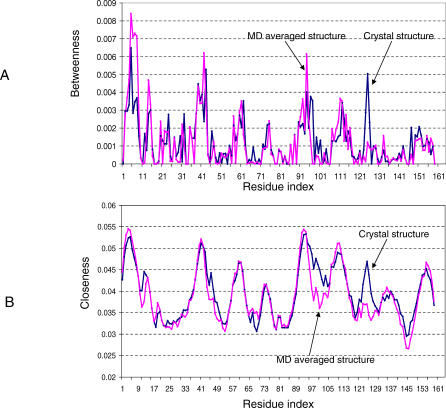
Comparison of Network Centrality of the Crystal Structure (Blue Line) and MD Average Structure (Pink Line) for the Native DHFR (A) Betweenness. (B) Closeness.

Interestingly, even though there is a large conformational change for loop 10–23, the changes of the network centrality around the loop are much smaller than in the region of residues 120–125, which has only small geometrical variation. Therefore, the analyses of both the static crystal structure and the MD average native structure have shown a consistent functional role for the Met20 loop of residues 10–23.

Comparison with the native DHFR structures (both crystal structure and MD average structure) shows that the network centrality of circular permutation variants change considerably. The betweenness changes are random-like and can draw different patterns for the folding element group and the nonfolding element group (unpublished data). When we look at the closeness centrality, we see that the cuttings in the folding element group cause larger deviation in the binding-related regions compared with the native DHFR structures. Figure 6 plots two graphs to compare the changes of the closeness for the two cutting groups. In Figure 6A, the blue line is the z-score for the MD wild-ave structure, the pink line is the average z-score for the folding element cutting group, and the green line is the average z-score for the nonfolding element cutting group. It can be seen that the three lines have very similar shapes, indicating overall consistency in the network analysis. In Figure 6B, we plot the changes of the z-scores for the two cutting groups, comparing with the z-score of the closeness for the MD wild-ave structure. The larger the z-score change, the larger the perturbation from the permutation cutting.

**Figure 6:**
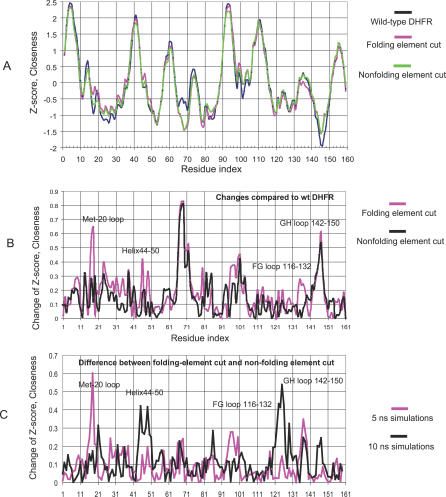
The Change of Closeness for MD Average Structures for Folding and Nonfolding Groups (A) z-Score of closeness for MD average wild-type DHFR structure (blue), averaged z-scores for the folding elements cutting group (pink), and averaged z-scores for the nonfolding elements cutting group (green). (B) The changes of the z-scores of the two cutting groups compared with the z-score of closeness for the MD average wild-type DHFR structure. (C) The differences of the z-scores of the folding element cutting group (a01, a02, a08, a08, and a10) and nonfolding element variants (b01, b04, b05, b08, and b09) averaged from 5-ns MD simulations and 10-ns simulations. The dynamics effects are in the functional loop regions.

At a first glance, we can see that there is a larger change around residues 10–50 for the folding element cutting group. This region has the Met-20 loop, and the helix 44–50, which is also in the binding site. In another catalytically important loop region (FG loop 116–132), we also see a larger change in the folding element cutting group. Both cutting groups have similar perturbations in the GH loop (140–150).

The changes in closeness for these loop regions are dynamic. Figure 6C shows the difference between the closeness of the folding element cutting group and nonfolding element cutting group, averaged from the 2.5- to 5.0-ns simulations and the 5.0- to 10.0-ns simulations. It can be seen that while the difference for the Met-20 loop is large during the 2.5- to 5.0-ns time span, the 5.0- to 10.0-ns dynamics show differences in the regions of helix 44–50 and the FG loop 116–132.

In summary, the closeness centrality in the network analysis indicates the differing behavior of the functional loop regions upon breaking the chain connection in folding element regions versus in nonfolding element regions.

### Conclusions

We have carried out a systematic network analysis and MD simulations of the native DHFR and 19 of its circularly permuted variants. Starting with the crystal structure of the native DHFR, we constructed circular permutations by linking the N-termini and C-termini with five glycine residues, and, following the experiment, introduced breaks in the chain connections in ten folding element regions and in nine nonfolding element regions. The 5-ns MD simulations provided average structures for the native DHFR and for the circularly permuted mutants that are able to fold according to experimental data [5]. For ten circularly permuted variants (five from the folding element group and five from the nonfolding element group), additional 5-ns simulations provided dynamics effects reflecting the changes in the closeness in the functional loop regions. Analysis of the structural variations illustrated that there are no distinctive structural features indicating whether particular circularly permuted mutants with cuttings within the folding elements will be able to fold. Simulations of experimentally determined permuted mutants that are unable to fold sample only the local minimum on the protein-folding energy landscape and do not reveal the true folding properties of such circularly permuted variants. Nevertheless, our combined MD and network studies with breaks in the chain connection in the folding element or nonfolding element regions present different patterns of perturbation of the network properties near the active site.

Our network analysis further leads us to propose that ligand binding induces network-bridging effects in the protein structure. We observed that substrate binding has profound effects on the DHFR network centrality, especially on closeness. The protein active site is near or coincides with residues through which the shortest paths in the residue interaction network tend to go. Our results suggest that cofactor binding leads to a modification of the interaction network, with most of the interactions now passing through the cofactor. The average shortest path is shorter with cofactor binding. Our findings are consistent with experimental observations that substrate binding increases DHFR folding stability [23,24].

In conclusion, our analysis demonstrates that active site dynamics of DHFR are communicated to the whole protein via both the peptide backbone and the nonbonded residue contacts. Such communication is indicated by the closeness change accompanying ligand binding, by breaking chain connections in the folding element region, and by breaking chain connections in the nonfolding element region. Even though the native and the circularly permuted proteins have similar overall folds, breaking the chain connections at different regions and ligand binding can change the properties of the network.

## Materials and Methods

### MD simulations.

The structure of DHFR from E. coli (closed state) was taken from the Protein Data Bank (http://www.rcsb.org/pdb). Both crystallographic waters and substrates were deleted. Circular permutation of the protein consists of connecting the native N- and C-termini covalently with a peptide linker and cleaving the peptide backbone at one specific site. Because a five-glycine peptide was shown to be the most favorable linker in the circularly permuted DHFR [5], this peptide linker was used in all variants in our study. The MOE software (Chemical Computing Group, http://www.chemcomp.com) was used to obtain all the circularly permuted variants of the native DHFR, which now has 164 residues. A total of 19 variants were selected for simulation: ten in the proposed folding element region and nine in the nonfolding element region (Table 1).

The CHARMM program [25] (version 30b1) was used for all computations with the CHARMM force field version 22 using all atom representation [26]. The native DHFR and its circularly permuted variants were simulated in a 60 × 60 × 60 Å^3^ explicitly solvated periodic box. TIP3P water molecules were introduced. The simulations were carried out with a distance cutoff of 13 Å and a constant dielectric constant of 1. Each simulation was initialized with adopted basis Newton-Raphson (ABNR) minimization followed by 3 ps system heating and 17 ps system equilibration. A production simulation run was carried out for each of the protein structures described above with a 1-fs time step. The coordinates were saved at 1-ps time intervals. Each simulation was run at 300 °K for 5 ns. The average structures from the last 2.5-ns simulations were used for network analysis.

### Molecular docking of DHFR ligand complex.

Patchdock [27], a geometry-based molecular docking algorithm, was used to generate clusters for the DHFR–ligand interactions. We kept those clusters with at least four docked conformations with RMSDs within 3.5 Å.

### Analysis of the protein residue interaction network: Chain connection and nonbonded packing.

The amino acid network is defined by all residues within a contact distance. Residue interaction network analysis often uses uniformed distance as long as the contacting residues are within a cutoff distance. Such an approach does not distinguish between chain connection and nonbonded interaction. A recent network permutation analysis of DHFR based on such a definition led to two separate regions [22].

The nature of the chemical bond argues for a strong communication when two residues are sequentially linked by a peptide bond. In Figure 7, we show that the most connected residue Ile5 has two bonded connections with Leu4 and Ala6 and the two closely packed residues Ile94 and Tyr111. Since residues connected by a peptide bond have shorter C_α_ distances, a weighting of distance can distinguish between chain connection and nonbonded interaction. Thus, we define contact distances based on the C_α_ of all residues within 6.0 Å and use the integer of the distance as weight. Therefore, 6 is coded for a distance of 6.0 Å, 5 for a distance between 5.0 and 6.0 Å, 4 for a distance between 4.0 and 5.0 Å, and 3 for a distance between 3.0 and 4.0 Å. The chain connection can have a distance index of 3 or 4, and a nonbonded contact can have a distance index of 5 or 6. This definition can effectively reflect the effects of chain cleavage on the network properties. The algorithm by Pape [28] was used to calculate the shortest path lengths between nodes. Two network properties are computed to characterize network properties of a given protein structure.

**Figure 7:**
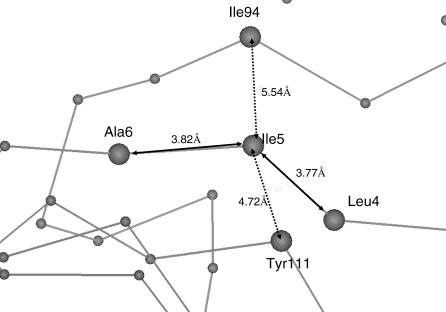
An Illustration of the Difference between Backbone-Connected Contact and Nonbonded Contact The peptide backbone-linked connections are shown as solid arrows, and the nonbonded packing is shown as dashed arrows.

The betweenness [29] is one of the standard measures of node centrality. The betweenness *bi* of a node *i* is defined as

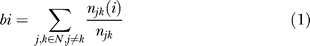
where *n_jk_* is the number of shortest paths connecting *j* and *k*, while *n_jk_(i)* is the number of shortest paths connecting *j* and *k* and passing through *i*.

The closeness centrality of node *x* is the inverse of the average distance between *x* and other nodes:

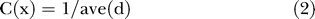



The z-score of the closeness is calculated by z-score = (C (x) − μ) / σ, where *μ* is the average value of closeness and *σ* is the standard deviation.

## Supporting Information

### Accession Numbers

The Protein Data Bank (http://www.rcsb.org/pdb) accession numbers for the structures discussed in this paper are open state (1ra9); closed state (1rx1); and occluded state (1rx5).

## Abbreviations

DHFR: dihydrofolate reductase
MD: molecular dynamics
RMSD: root mean square deviation
RMSF: root mean square fluctuation
wild-ave: average structure of wild-type DHFR
